# A Novel Offset Cancellation Based on Parasitic-Insensitive Switched-Capacitor Sensing Circuit for the Out-of-Plane Single-Gimbaled Decoupled CMOS-MEMS Gyroscope

**DOI:** 10.3390/s130303568

**Published:** 2013-03-14

**Authors:** Ming-Hui Chang, Han-Pang Huang

**Affiliations:** Department of Mechanical Engineering, National Taiwan University, Taipei 10617, Taiwan; E-Mail: d93522037@ntu.edu.tw

**Keywords:** out-of-plane gyroscope, parasitic-insensitive switched-capacitor (PISC), ΣΔ ADC

## Abstract

This paper presents a novel parasitic-insensitive switched-capacitor (PISC) sensing circuit design in order to obtain high sensitivity and ultra linearity and reduce the parasitic effect for the out-of-plane single-gimbaled decoupled CMOS-MEMS gyroscope (SGDG). According to the simulation results, the proposed PISC circuit has better sensitivity and high linearity in a wide dynamic range. Experimental results also show a better performance. In addition, the PISC circuit can use signal processing to cancel the offset and noise. Thus, this circuit is very suitable for gyroscope measurement.

## 1. Introduction

Generally speaking, MEMS gyroscopes only have relatively small detection capacitances; therefore, the sensing circuit design is very important. The sensing mechanisms of MEMS gyroscopes are mainly categorized into piezoresistive [1–5] and capacitive [6–13] mechanisms. Capacitive gyroscopes have simpler structure and hence lower fabrication cost. In addition, they provide lower power consumption, higher sensitivity, and higher reliability as well as lower nonlinearity, lower temperature dependency, lower noise, and lower drift. Capacitance is also easily available in silicon technology because it does not require special materials and process steps. On the other hand, the piezoresistive sensing scheme is widely used in accelerometers. If the gyroscopes use piezoresistive materials, they should be driven in their primary modes with different mechanism because piezoresistive materials cannot induce passive nature by themselves [14]. In addition, its sensitivity is low, and its temperature dependency is high compared to the capacitive gyroscope. Hence, it is mainly used in low-end products.

Capacitive sensing circuits are categorized into synchronous [15], switched-capacitor [12,16–18], and fully differential circuits [8,11,19,20]. The transfer functions of the first two are independent of parasitic capacitance, and they can cancel the flicker noise and DC offset voltage to provide high accuracy. The synchronous sensing circuit requires bias control with either a large value off-chip or large-area integrated resistive circuit component [15]. In addition, in the frequency response, the transfer function of the synchronous sensing circuit has a zero point that increases the phase delay so that it cannot control feedback. The architecture of the fully differential sensing circuit is simple and contains no charge cancellation error, but its signals are attenuated by parasitic capacitance [19]. The switched-capacitor circuit technique is based on the realization that the capacitor and switch are equivalent to a resistor for reducing the layout area [16–18]. This technique can realize a variety of other signal-processing blocks, such as gain-stage, voltage-controlled oscillators (VCOs), and modulators. The switched-capacitor circuit can provide stability but it reduces the open-loop gain. In addition, the switched-capacitor circuit provides a virtual ground and robust DC biasing at the sensing node so that the sensed signal is insensitive to parasitic capacitance and undesirable charging. This circuit also offers a wide range of techniques to suppress offset and low-frequency noise, such as correlated double sampling (CDS) and programmable capacitor array (PCA). Thus, this circuit is very suitable for gyroscope measurement. A novel parasitic-insensitive switched-capacitor (PISC) sensing circuit is designed in order to get high sensitivity and ultra linearity and reduce the parasitic effect for the out-of-plane single-gimbaled decoupled gyroscope (SGDG) in this study.

This paper is organized as follows: in Section 2, the PISC circuit design is briefly introduced. The simulation results are presented in Section 3. The experimental results are presented in Section 4. Finally, the conclusions are given in Section 5.

## 2. Circuit Design

**Figure 1.**
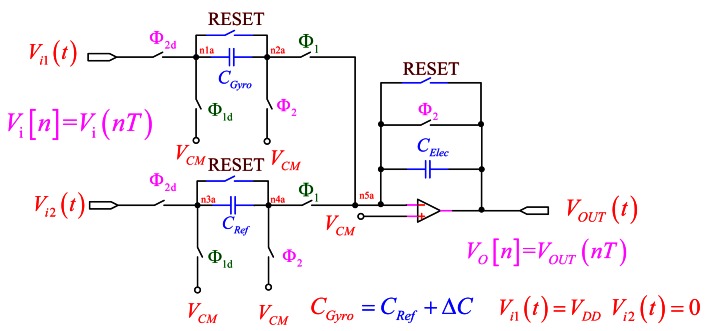
The PISC sensing circuit.

The on-chip sensing circuit uses the switched-capacitor sensing circuit that detects the capacitance change due to the deflection of the proof mass. During the sampling phase of a traditional switched-capacitor amplifier, it only samples the input, sets the output to zero, and provides no amplification. This raises concern about its stability, and reduces the open-loop gain. The capacitors used are known as double-poly capacitors. There also exists a substantial parasitic capacitance. In order to reduce the parasitic effect, we propose the PISC sensing circuit, as shown in Figure 1 [16,17]. Here, the “RESET” switches are only utilized for discharging the capacitors initially. The *C_Gyro_* is capacitance of detection of the gyroscope, the *C_Ref_* is initial capacitance of detection of the gyroscope and the *C_Elec_* is amplification capacitance. *V_CM_* is common mode voltage, and its value is *V_DD_*/2. In this study, the *V_CM_* is set to 1.65 V.

At the beginning, the input signals voltages of the circuit are +1.65 V and −1.65 V. In order to maintain the cut-off condition for all the substrate-to-channel junctions, the substrate is usually connected to the most negative power supply in an NMOS circuit (the most positive in a PMOS circuit) [18]. Thus, we use the concept of voltage translation to change the input signal voltage. The input signal *V_i_*_1_(*t*) changes to *V_DD_*, and the other *V_i_*_2_(*t*) changes to zero.

**Figure 2.**
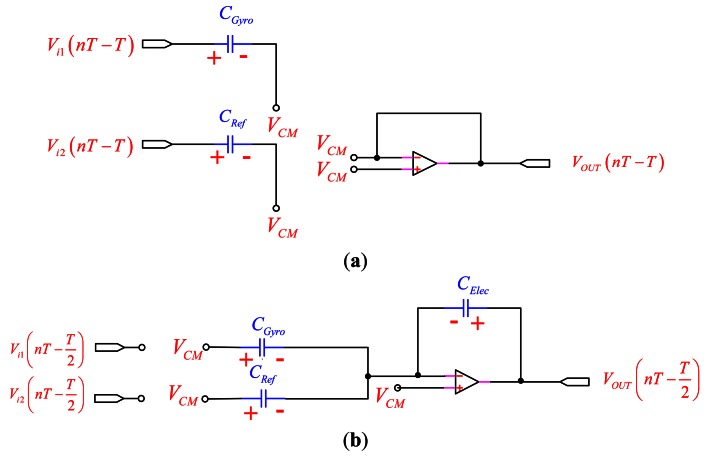
The PISC sensing circuit for the two operations: (**a**) sampling mode; (**b**) amplification mode.

As shown in Figure 2(a), in the sampling mode, *Φ*_2_ is on and *Φ*_1_ is off, allowing the voltage across *C_Gyro_* and *C_Ref_* to track *V_i_*_1_(*t*) and *V_i_*_2_(*t*) while the operational amplifier (OP-amp) and *C_Elec_* hold the previous value. In the transition to the amplification mode, the charge stored on *C_Gyro_* and *C_Ref_* are transferred to *C_Elec_* through the virtual ground node, as shown in Figure 2(b).

The results in the transfer function for this switched-capacitor circuit are given by:

(1) $$\frac{V_{o}(z)}{V_{i1}(z)}=\frac{1}{2}\left(\frac{-C_{\text{Ref}}+C_{\text{Gyro}}}{C_{\text{Elec}}}\right)z^{-\frac{1}{2}}=\frac{1}{2}\left(\frac{\Delta C}{C_{\text{Elec}}}\right)z^{-\frac{1}{2}}$$

**Figure 3.**
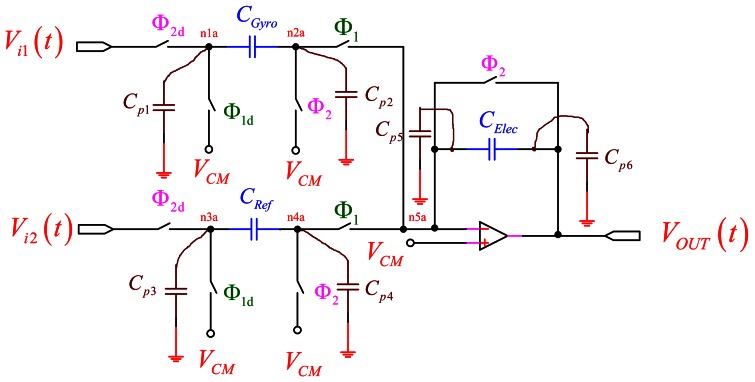
The PISC sensing circuit with parasitic capacitances shown.

Note that Equation (1) represents its gain coefficient as the ratio of the difference capacitance and *C_Elec_*. The z^−1/2^ represents a period delay and can be ignored [16]. The behavior of the circuit with respect to parasitic capacitances is plotted in Figure 3 [16].

Here, *C_p_*_1_ is continuously being charged to *V_i_*_1_(*t*) and discharged to the common mode (*V_CM_*). However, when *Φ*_2_ is turned on, the fact that *C_p_*_1_ is also charged to *V_i_*_1_(*t* − 1) does not affect the charge that is placed on *C_Gyro_*. When *Φ*_1_ is turned on, *C_p_*_1_ is discharged through the *Φ*_1_ switch attached to its node, and none of the discharging current passes through *C_Gyro_* to affect the charge accumulating on *C_Elec_*. Therefore, it does not affect the circuit operation. In addition, *C_p_*_2_ is either connected to *V_CM_* through the *Φ*_2_ switch or to the virtual ground through the *Φ*_1_ switch. Since *C_p_*_2_ always remains discharged, it does not affect the operation of the circuit. Similarly, the operations of *C_p_*_3_ and *C_p_*_4_ do not effect as *C_p_*_1_ and *C_p_*_2_. The effect of *C_p_*_5_ on the transfer function is small since it is always connected to the virtual ground of the OP-amp. Finally, *C_p_*_6_ is connected to the OP-amp output. Although it may affect the speed of the OP-amp, it would not affect the final settling point of the OP-amp output.

**Figure 4.**
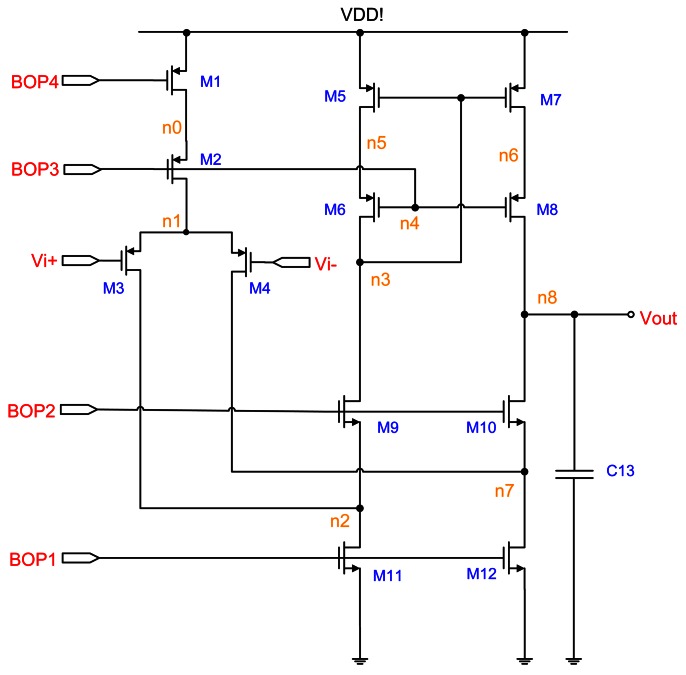
Schematic of the OP-amp circuit.

A switched-capacitor circuit is realized with basic building blocks such as the OP-amp, capacitors, switches, and non-overlapping clocks. The OP-amp circuit is shown in Figure 4. To improve the circuit stability, the OP-amp must provide a high enough stability margin. The folded-cascode OP-amp architecture is selected to provide the phase margin of 78.906°. The input differential pairs use large devices with a parallel symmetric layout for good matching, thereby reducing the input voltage offset and flicker (1/*f*) noise. To increase the phase margin of OP-amp, a large output compensation capacitor of up to 3.8 pF is used; it is made of double poly (poly1 and poly2). Table 1 provides a list of the device sizes.

**Figure 5.**
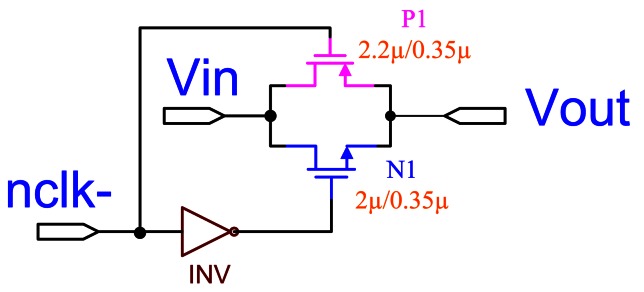
Transmission gate.

**Figure 6.**
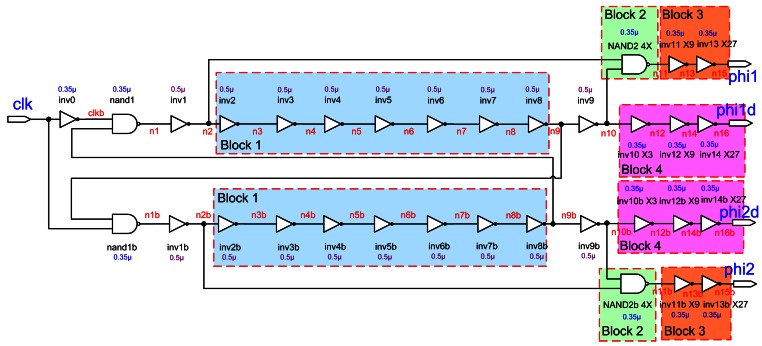
Schematic of the non-overlapping clock circuit.

The requirements for switches used in this paper are that they have very high off resistance and low on resistance, and introduce no offset voltage when turned on. In order to achieve the full signal range of 0 to 3.3 V, we used a negative triggered CMOS transmission gate, as shown in Figure 5 [16]. To guarantee that the charge is not inadvertently lost, we adopt the non-overlapping clock, as shown in Figure 6, which is used to eliminate the clock feed-through influence [16,17]. It has two logic signals running at the same frequency and is changed in such a way that at no time are both signals high.

**Figure 7.**
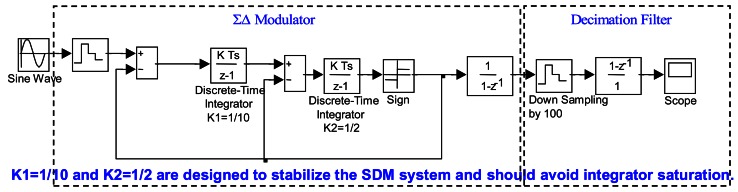
ΣΔ modulator behavior simulation.

A sensing circuit usually requires an ADC. In general, the output voltage for the sensing circuit is weak and small. A high resolution ADC is necessary for acquiring such a feeble signal. The ΣΔ ADC is the best choice for this application. It uses the over-sampling principle, noise shaping and decimation filtering to achieve the high resolution. It consists of a ΣΔ modulator and a decimation filter. In addition, the ΣΔ ADC operates in the discrete-time domain and is implemented using the switch-capacitor circuit technique. The switch-capacitor circuit can only be realized in MOS devices, not in bipolar transistors. In other words, the sensing circuit fabricated in the digital CMOS process is suitable for generating the digital output using ΣΔ ADCs. The behavior simulation model shown in Figure 7 is adopted for the ΣΔ modulator design to avoid the integrator overload and to satisfy the system requirements. The design is the second order ΣΔ modulator with a one-bit quantizer. The decimation digital filter is implemented by down-sampling and a comb filter. The discrete-time integrator parameters of K1 and K2 for the ΣΔ modulator are 0.1 and 0.5, respectively.

**Figure 8.**
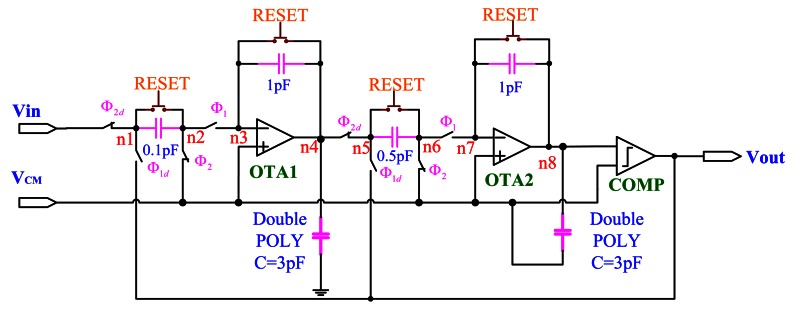
One-bit ΣΔ modulator circuit design.

**Figure 9.**
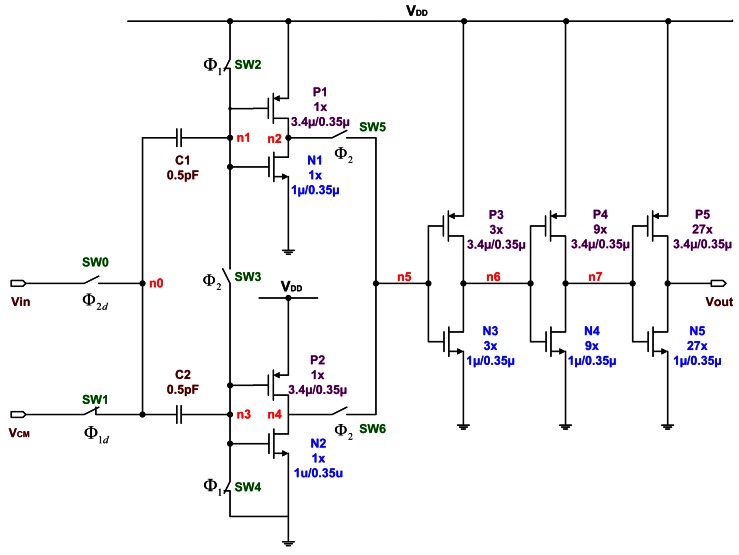
Comparator circuit design.

The circuit design for the ΣΔ modulator is shown in Figure 8. Here, “OTA1” and “OTA2” are folded-cascode OP-amps, as shown in Figure 4. The “COMP” indicates the comparator circuit, as shown in Figure 9. The “RESET” switches are only utilized for discharging the capacitors initially. The circuit is implemented using the switch-capacitor technique and controlled by a non-overlap clock circuit, as shown in Figure 6, which is used to eliminate the clock feed-through influence.

## 3. Simulation Results

In this paper, the simulation results for circuit design are the post-layout simulation results.

### 3.1. C_Ref_ Selection

**Figure 10.**
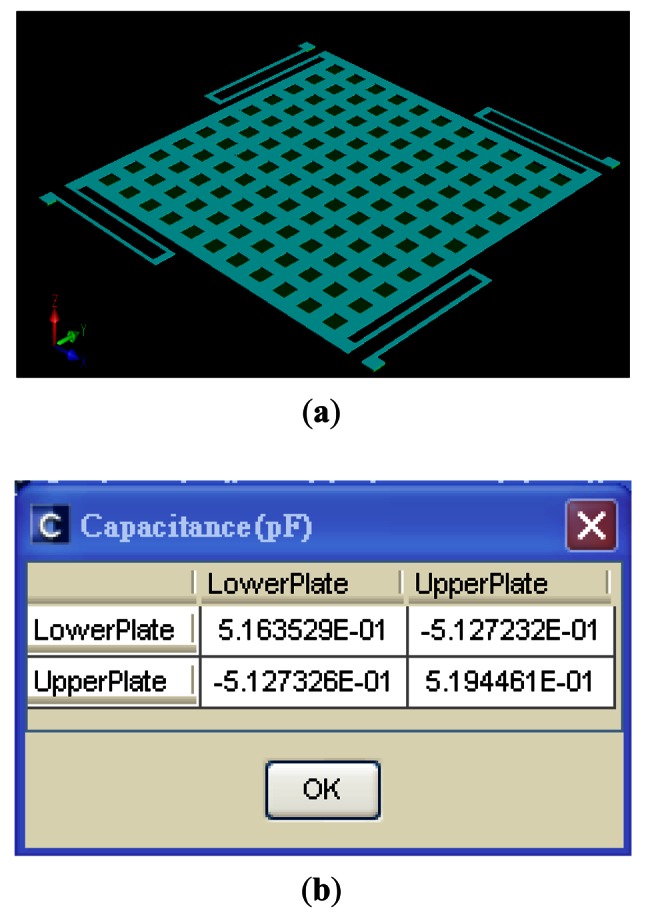
Using the CoventorWare simulator to solve *C_Ref_* (**a**) Solid model of the gyroscope. (**b**) Simulation results.

**Figure 11.**
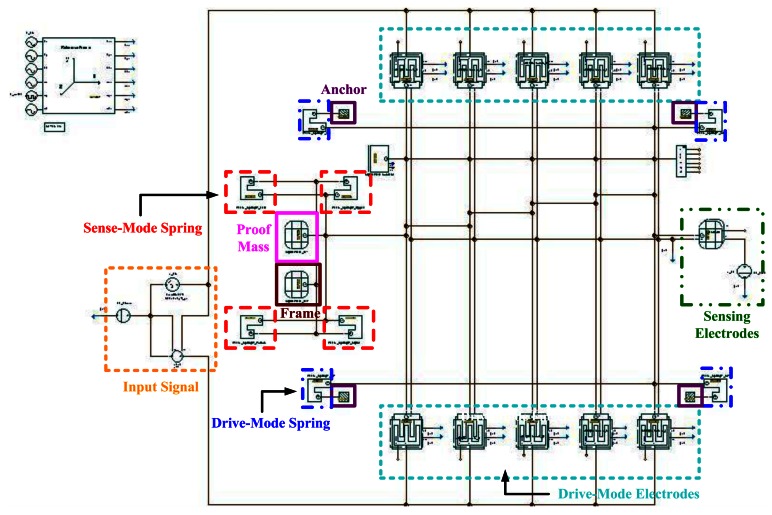
Schematic of out-of-plane SGDG.

**Figure 12.**
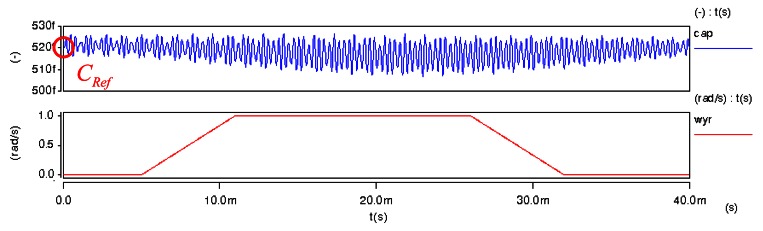
Using the ARCHITECT™ simulator to solve *C_Ref_*.

The *C_Ref_* is the initial capacitance of detection for the gyroscope. The capacitance value is simulated using the CoventorWare FEM simulator. The simulator uses the solid model of the gyroscope (see Figure 10(a)) and its capacitance value is about 516 fF, as shown in Figure 10(b). In addition, we also use the ARCHITECT™ simulator to estimate *C_Ref_*. It models the mechanical and electrical effects of these rough environments on the out-of-plane SGDG. In ARCHITECT™, we work in a schematic-driven environment using symbols to represent individual components or elements of components, as seen in Figure 11. Typically, these component models are parameterized. The simulation result of electrode capacitance (*cap*) for the transient analysis is shown in Figure 12. It can be found that the initial capacitance, *C_Ref_* is about 519 fF when the sensing signal (*wyr*) is 1 rad/s. The capacitance is calculated to be about 499 fF by the formula. After comparing the three methods, we choose this value of about 500 fF for a convenient layout.

### 3.2. C_Elec_ Selection

In Table 2, the output voltage of *C_Gyro_* = 500 fF is close to *V_CM_* (= 1.65 V) when *C_Elec_* is 60 fF. Here, these output voltages are simulated by the HSPICE simulator. However, these values should be actually approached when *C_Elec_* is 63 fF. But this time the layout is poor to achieve. Therefore, we choose *C_Elec_* to be 60 fF.

### 3.3. Parasitic-Insensitive Switched-Capacitor Circuit

**Figure 13.**
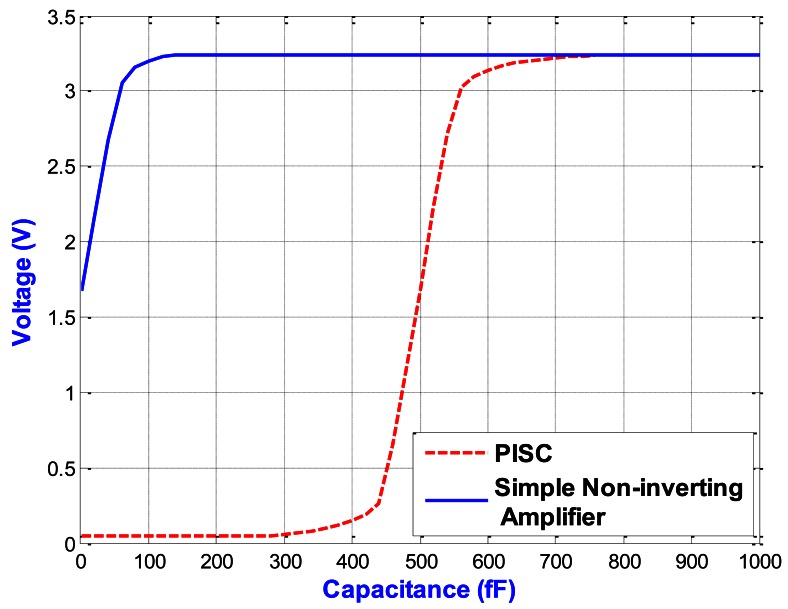
C-V curves of the two capacitive sensing circuits.

**Figure 14.**
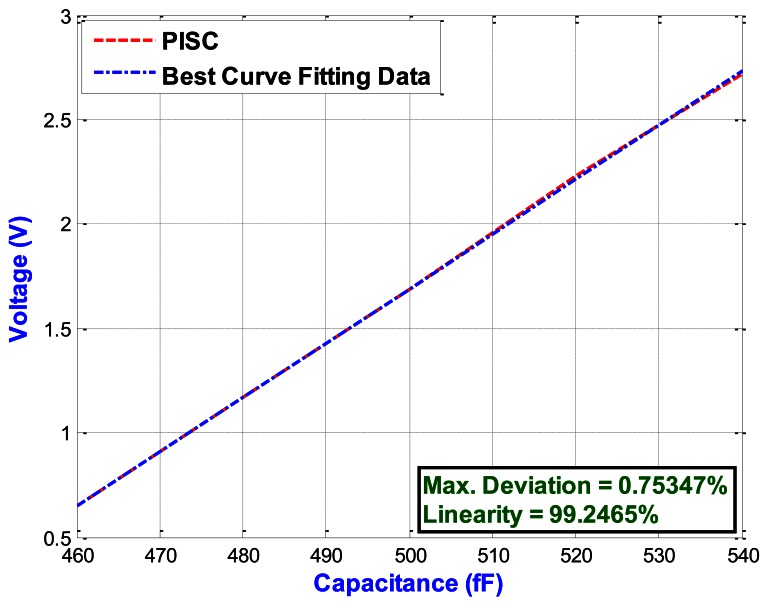
Enlarge picture of the PISC circuit of Figure 13 (460 fF–540 fF).

Figure 13 shows the C-V curves of the two capacitive sensing circuits, including the PISC circuit and simple non-inverting amplifier. The proposed PISC circuit has the higher dynamic voltage output and sensing range than those of the simple non-inverting circuit. Figure 14 illustrates the enlarging parts of the PISC circuit of Figure 13 between 460 fF and 540 fF.

The proposed PISC circuit has an ultra linearity of 99.2465%. The detailed specifications and comparisons of the two circuits are summarized in Table 3. It can be noted that the proposed PISC circuit has the higher capacitance sensitivity, lower power dissipation, and smaller noise floor, but its resolution and voltage offset are less than those of the simple non-inverting circuit. The main reason is that it uses more components than the simple non-inverting circuit.

**Figure 15.**
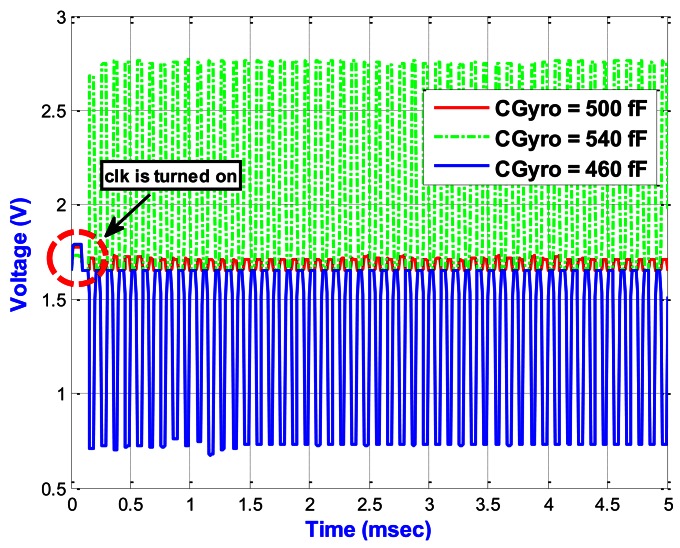
Output results of the proposed PISC circuit under different *C_Gyro_* (*C_Ref_* = 500 fF, *C_Elec_* = 60 fF).

**Figure 16.**
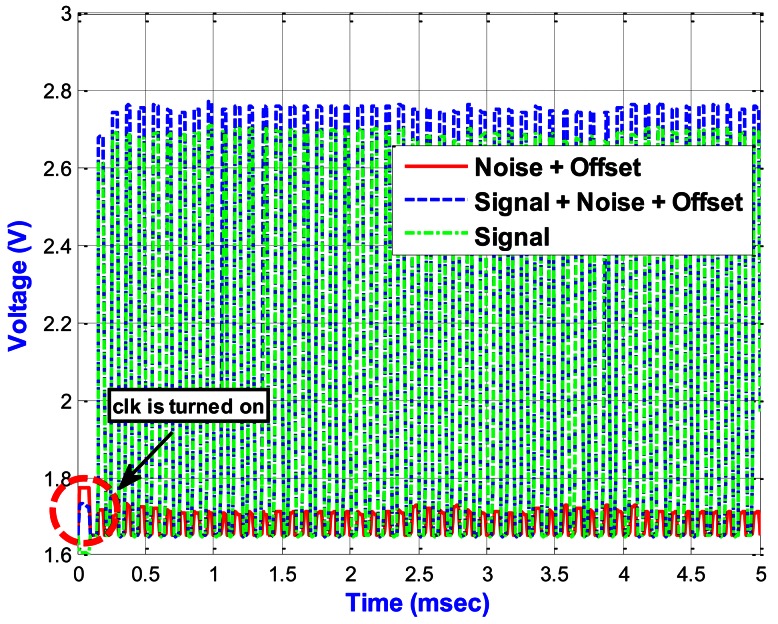
Output voltage using signal processing.

Figure 15 shows the output results of the proposed PISC circuit under different *C_Gyro_*. When the time reaches 100 μs, the *clk* in Figure 6 is turned on. From the simulation results, we can see that the proposed PISC circuit starts sampling after *clk* is turned on. In addition, the PISC circuit with *C_Gyro_* = 500 fF is mainly for the noise and offset signals. Thus, we can use signal processing to cancel the noise and offset, as shown in Figure 16.

Table 4 shows that the PISC circuit using signal processing has significantly smaller voltage offset and noise floor than those without using signal processing. Their improvement factors are 24.6% and 29.3%, respectively, compared to without using signal processing.

### 3.4. SDM (Sigma-Delta Modulator)

**Figure 17.**
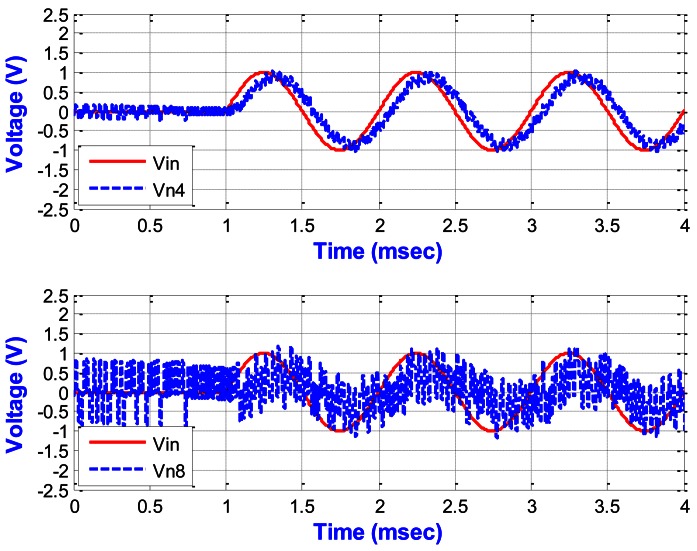
The simulation results of the SDM circuit from nodes n4 and n8 in Figure 8.

The simulation results of the SDM circuit simulated by HSPICE simulator from nodes of n4 and n8 in Figure 8 are shown in Figure 17 with a sinusoidal input.

Compared with the behavior simulation shown in Figure 7, they are almost the same. The bit-stream output of the ΣΔ modulator from the one-bit quantizer will be sent to the decimation filter to obtain the digital output.

### 3.5. Application (Combined Out-of-Plane SGDG in [21])

**Figure 18.**
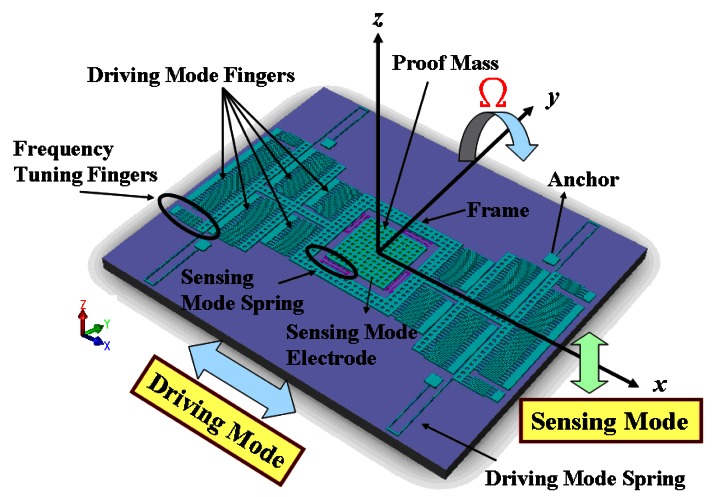
Out-of-plane SGDG.

A single-gimbaled configuration is used to decouple the drive and sense modes of the gyroscope. Figure 18 shows the topology of the SGDG, which is a *y*-axis sensitive out-of-plane gyroscope. The gyroscope is driven in the drive-mode with three rows of symmetrical comb fingers. The main purpose is to increase the number of fingers to 206 pairs (see Figure 18), and to provide greater driving force, the amplitude of the driving displacement, and less thermo-mechanical noise. As depicted in Figure 18, four translational springs outside the frame constrain the degree-of-freedom (DOF), and hence the frame can be driven only along the *x*-axis. Inside the frame, another set of linear springs allows the proof mass to move solely along the *z*-axis. When an external angular rate is applied to the proof mass along the *y*-axis, the proof mass vibrates due to Coriolis force along the *z*-axis. The Coriolis force is sensed by four inner springs.

**Figure 19.**
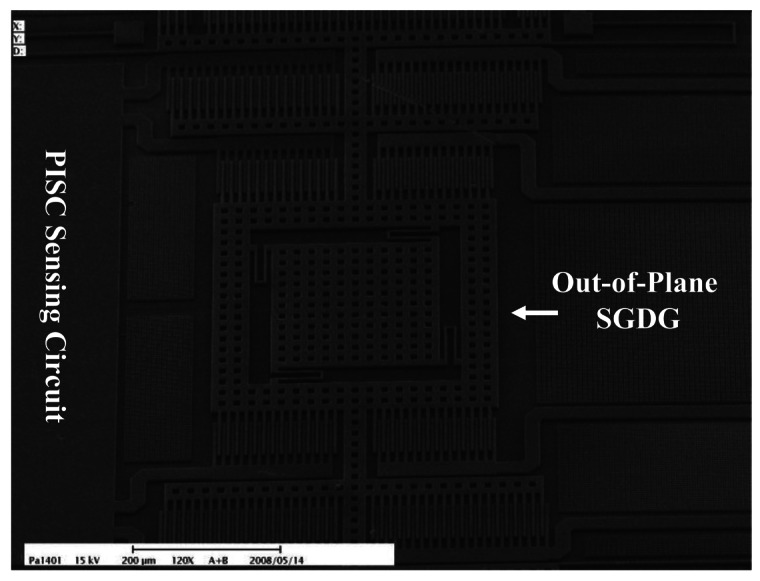
Top view of the out-of-plane SGDG.

The frame and the proof mass are actuated by the electrostatic comb drives at the resonant frequency. The sense electrode under the proof mass detects the capacitance difference using the parallel plates. The gyroscope is designed as a symmetrical and decoupled gyroscope in order to achieve a high sensitivity. The springs are all identical in shape in two modes, the drive and sense modes, and have different mass values. However, due to spring constant difference in the drive and sense modes, and in order to cancel process variations in the resonant frequencies, the drive-mode has two symmetrical frequency tuning fingers. These fingers create the force in the drive-mode and may reduce the frequency in some ranges relative to the applied DC voltage. The top view of the gyroscope chip, shown in Figure 19, is 1.725 mm × 1.515 mm and contains the SGDG and PISC blocks. The PISC circuit is located on the left of Figure 19. To avoid the damage caused by etching silicon dioxide in the wet etch, the PISC circuit is placed 50 μm away from the out-of-plane SGDG. Except for the microstructure, the whole chip area is covered by the top metal layer, M4.

**Figure 20.**
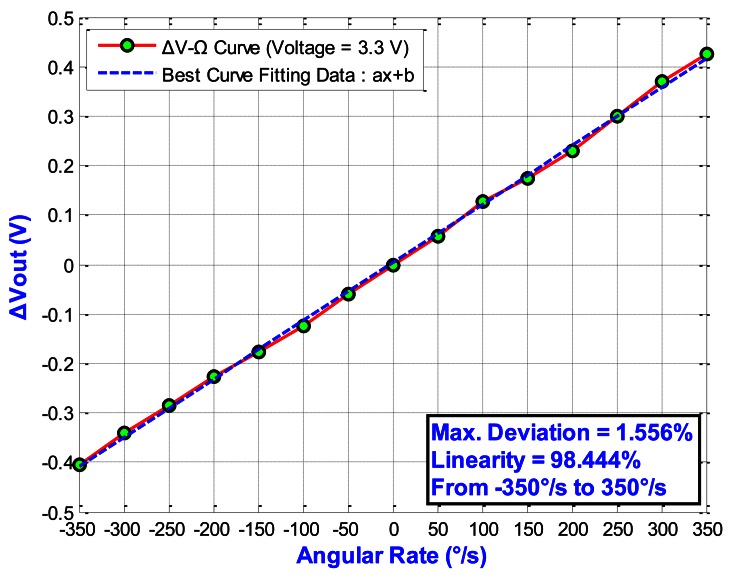
Output voltage of the gyroscope under different angular rates.

**Figure 21.**
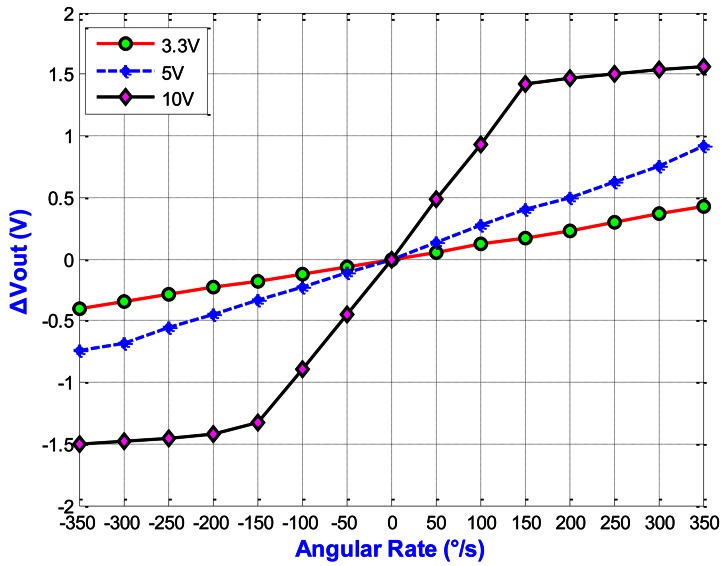
Output voltage *versus* the angular rate under different *V_Driving_*.

Figure 20 shows the output voltage of the gyroscope according to angular rate (*V_Driving_* = 3.3 V). Here, the *V_Driving_* means the driving voltage of the driving mode fingers. The sensitivity is 1.1866 mV/°/s and the linearity is 98.44% (with a full scale of 700 °/s). Figure 21 shows the output voltage *versus* the angular rate under different *V_Driving_*. For *V_Driving_* = 10 V, the sensitivity is 9.16 mV/°/s and the linearity is 99.16% (with a full scale of 300 °/s). The results of the out-of-plane gyroscope are summarized in Table 5.

## 4. Experimental Results

**Figure 22.**
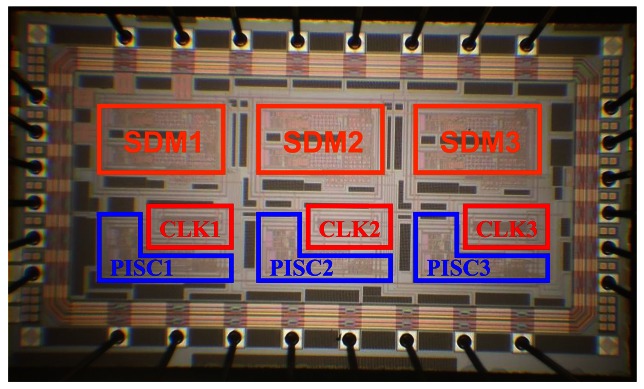
SEM of the proposed sensing circuit.

The proposed PISC circuit combined one-bit SDM circuit has been tested. The chip, shown in Figure 22, is 2.817 mm × 1.513 mm, and contains three PISCs, three CLKs, and three SDM blocks.

**Figure 23.**
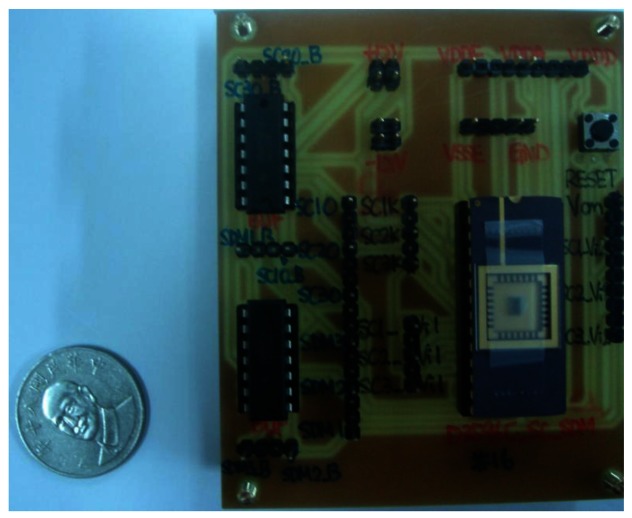
Photograph of the test PCB.

**Figure 24.**
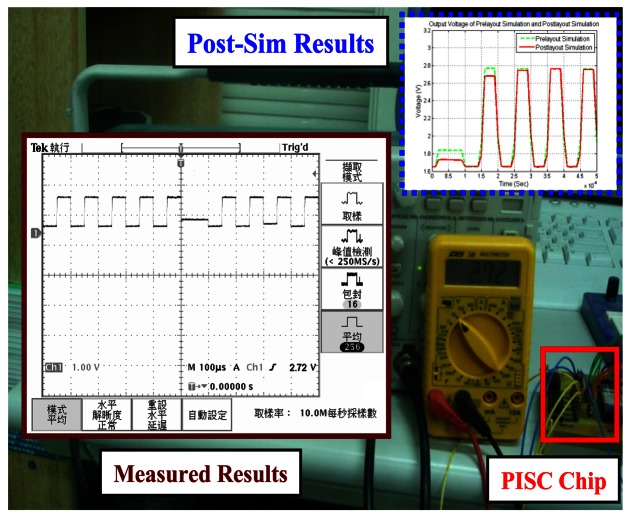
Measured results for PISC2 (*C_Gyro_* = 540 fF).

**Figure 25.**
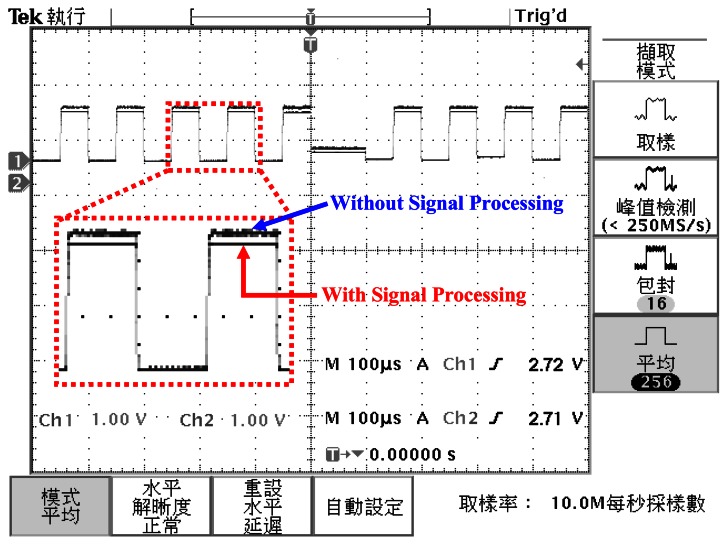
Experimental results using signal processing.

Here, the PISC1 means the PISC circuit has the *C_Gyro_* = 500 fF, the PISC2 means the PISC circuit has the *C_Gyro_* = 540 fF, and the PISC3 means the PISC circuit has the *C_Gyro_* = 460 fF. CLK indicates the non-overlapping clock circuit. A photograph of the test PCB block is shown in Figure 23. The measured voltage result for the PISC2 circuit (*C_Gyro_* = 540 fF) is 2.72 V, as shown in Figure 24. The pre- and post-layout simulation results are shown in the up-right corner in Figure 24. These results are very similar. Other results are summarized in Table 6. The experimental results of the PISC circuit using signal processing have significantly smaller voltage offset and noise floor than those without using signal processing, as shown in Figure 25.

Table 7 summarizes a comparison on the results of the proposed PISC circuit and other capacitive sensing circuits reported in the literature. It can be noted that the proposed PISC circuit has the smallest noise floor and chip area. The voltage offset is comparable to [11] but smaller than the remaining solutions listed in Table 7. The capacitive sensing circuits in [11] is simulation result. The resolution is comparable to [12] but smaller than other capacitive sensing circuits already presented in the literature. For standalone applications, the device gives an analog output and provides digital output with embedded one-bit ΣΔ ADC.

## 5. Conclusions

In this study, we present a novel PISC sensing circuit design in order to obtain high sensitivity and ultra linearity for an out-of-plane gyroscope. From the simulation results, we can see that the proposed PISC circuit has a good sensitivity of 9.16 mV/°/s and a high linearity of 99.16% in a wide dynamic range from −300 °/s to 300 °/s. The experimental results also show a good performance. In addition, the PISC circuit can use signal processing to cancel the offset and noise. Thus, this circuit is very suitable for gyroscope measurement.

## Figures and Tables

**Table 1.** Device sizes used in Figure 4.

**OP-amp**		**Sizes**
	
**Components**	**Type**	**transistor: width/length/# of fingers capacitor: width/length/# of parallel/value**
M1	PMOS	40 μm/1 μm/2
M2	PMOS	40 μm/1 μm/2
M3	PMOS	40 μm/2 μm/4
M4	PMOS	40 μm/2 μm/4
M5	PMOS	40 μm/2 μm/2
M6	PMOS	40 μm/2 μm/2
M7	PMOS	40 μm/2 μm/2
M8	PMOS	40 μm/2 μm/2
M9	NMOS	20 μm/1 μm/1
M10	NMOS	20 μm/1 μm/1
M11	NMOS	20 μm/2 μm/2
M12	NMOS	20 μm/2 μm/2
C13	PIP	2.1 μm/31.93 μm/9/3.8 pF

**Table 2.** Output voltage (unit: V) under different *C_Gyro_* (*C_Elec_* swept from 10 fF to 100 fF).

***C****_Gyro_***(fF)**	***C****_Elec_***(fF)**									

	**10**	**20**	**30**	**40**	**50**	**60**	**70**	**80**	**90**	**100**	
460	0.18	0.203	0.235	0.308	0.488	0.661	0.79	0.889	0.994	1.032
500	1.88	1.808	1.748	1.730	1.722	1.691	1.71	1.692	1.693	1.685
540	3.12	3.104	3.050	2.982	2.830	2.688	2.55	2.454	2.380	2.305

**Table 3.** Comparison of the two circuits (*C_Gyro_* = 540 fF (25 °C), Frequency = 10 kHz).

**Term**	**PISC**	**Simple Non-inverting Amplifier**
Supply Voltage (V)	3.3	3.3
Power Dissipation (mW)	2.4108	2.4392
Dynamic Voltage Output (V)	0.0445–3.2427	1.6752–3.2434
ΔC Range (fF)	±40	0–40
Sensing Range (fF)	440–540	0–40
Capacitance Sensitivity (mV/fF)	25.8938	24.9875
Linearity (%)	99.2465	98.5426
Ideal Resolution (fF)	0.01273	0.00962
Voltage Offset (mV)	1.9964	1.5534
Noise Floor (μV/Hz^1/2^) (at 10 kHz)	0.1564	4.6716

**Table 4.** Simulated demonstration of performance improvement with signal processing.

**Term**	**Without Signal Processing**	**With Signal Processing**	**Improvement Factor (%)**
Voltage Offset (mV)	1.9964	1.5053	24.6
Noise Floor (μV/Hz^1/2^) (at 10 kHz)	0.1564	0.1106	29.3

**Table 5.** Specification of the out-of-plane gyroscope.

**Parameters**	**3.3 V**	**5 V**	**10 V**
Sensitivity (mV/°/s)	1.1866	2.3882	9.16
Dynamic Range (°/s) (Full Scale)	700	600	300
Linearity (%)	98.44	98.28	99.16

**Table 6.** Comparison of simulation and experimental results for PISC circuits.

**Term**	**Simulation Results**	**Experimental Results**
Supply Voltage (V)	3.3	3.3
PISC1 (V)	1.65–1.78	1.6–1.81
PISC2 (V)	1.65–2.78	1.6–2.8
PISC3 (V)	0.7–1.65	0.7–1.6

**Table 7.** Comparison with CMOS capacitive sensing circuits in the literature.

	**[7]**	**[10]**	**[11]**	**[12]**	**[13]**	**[19]**	**This Work**
Process	0.35 μm, CMOS	0.8 μm, CMOS	0.35 μm, CMOS	2 μm, CMOS	0.35 μm, CMOS	0.8 μm, CMOS	0.35 μm, CMOS
Application	Gyroscope	Accelerometer	Accelerometer	Accelerometer	Gyroscope	Transducer	Gyroscope
Architecture	Fully Differential	Fully Differential	Fully Differential	Switched Capacitor	DC Sensing	Differential	PISC
ADC	7 bits SAR	None	1 bit ΣΔ	None	None	None	1 bit ΣΔ
Sensitivity	N.A.	4 fF/m/s^2^	N.A.	0.122 V/m/s^2^	N.A.	40 mV/fF	9.16 mV/°/s
Linearity (%)	N.A.	N.A.	N.A.	99.95	N.A.	N.A.	99.16
ΔC Range (fF)	∼400	N.A.	0.13	∼1,800	N.A.	0.2	±40
Capacitance Sensitivity (mV/fF)	N.A.	N.A.	∼10	10 ∼ 50	N.A.	42	25.8938
Resolution (fF)	3.5	0.0255	N.A.	<0.01	0.195	10	0.01273
Voltage Offset (mV)	8	N.A.	1	N.A.	N.A.	200	1.9964
Noise Floor (μV/Hz^1/2^)	N.A.	37 at 36 kHz	0.2 at 500 kHz	0.96 at 1 kHz	N.A.	N.A.	0.1564 at 10 kHz
Power Dissipation (mW)	N.A.	N.A.	5 at 3 V	N.A.	N.A.	N.A.	2.2973 at 3.3 V
Chip Area (mm^2^)	3.625	5.76	N.A.	15.2	1.44	14.62	1.421
Simulation/Chip	C	C	S	C	C	C	C
